# Recent Syntheses of 1,2,3,4-Tetrahydroquinolines, 2,3-Dihydro-4(1*H*)-quinolinones and 4(1*H*)-Quinolinones using Domino Reactions

**DOI:** 10.3390/molecules19010204

**Published:** 2013-12-24

**Authors:** Baskar Nammalwar, Richard A. Bunce

**Affiliations:** Department of Chemistry, Oklahoma State University, Stillwater, OK 74078-3071, USA

**Keywords:** domino reaction, tandem reaction, cascade reaction, 1,2,3,4-tetrahydroquinoline, 2,3-dihydro-4(1*H*)-quinolinone, 4(1*H*)-quinolinone, heterocycle synthesis, drug synthesis

## Abstract

A review of the recent literature is given focusing on synthetic approaches to 1,2,3,4-tetrahydroquinolines, 2,3-dihydro-4(1*H*)-quinolinones and 4(1*H*)-quinolinones using domino reactions. These syntheses involve: (1) reduction or oxidation followed by cyclization; (2) S_N_Ar-terminated sequences; (3) acid-catalyzed ring closures or rearrangements; (4) high temperature cyclizations and (5) metal-promoted processes as well as several less thoroughly studied reactions. Each domino method is presented with a brief discussion of mechanism, scope, yields, simplicity and potential utility.

## 1. Introduction

Domino reactions, also known as tandem or cascade reactions, have emerged as a highly effective strategy for the synthesis of bioactive natural products and pharmaceutical agents [1]. These methods enable chemists to perform complex synthetic conversions with high efficiency using simple starting materials, often via a biomimetic pathway [2]. Thus, domino reactions contribute immensely to synthetic drug design strategies, enhance aesthetic approaches in total synthesis, and improve yields in large-scale synthesis [1,2]. The advantages of these methods include excellent atom economy, high selectivity, and less waste [3,4]. Additionally, using these strategies, multiple transformations can be carried out in a single laboratory operation without the isolation of intermediates making them prime examples of green chemistry [5,6]. Despite the widespread proliferation of domino reactions, researchers have continued to channel their efforts in this area as new structures with chiral architectures and novel substitution patterns are required. 

The goal of this survey is to provide readers with a summary and critical evaluation of the domino strategy as it pertains to the synthesis of tetrahydroquinolines, 2,3-dihydro-4(*1H*)-quinolinones and 4(*1H*)-quinolinones. We do not seek to provide a comprehensive treatise on methods to generate these heterocyclic scaffolds but rather to highlight the application of domino strategies for their preparation. Other, more expansive, reviews have appeared covering the vast methodology developed to assemble these systems [5,6], but our focus here is only on recently reported one-flask, multistep processes. A review has recently been published on cascade reactions to prepare heterocycles [7], but it focused primarily on protocols to prepare indoles, with only minimal coverage of the derivatives described in this document.

Domino approaches to the construction of the title compounds can be broadly classified into five different categories: (1) reduction or oxidation followed by cyclization [8,9]; (2) S_N_Ar-terminated sequences [10]; (3) acid-catalyzed ring closures or rearrangements [11,12]; (4) high temperature cyclizations [13,14,15]; or (5) metal-promoted processes [16,17,18]. These are only the major strategies that will be addressed. Several less thoroughly studied transformations are also included, and all of the methods will be discussed in terms of mechanism, scope, yields, simplicity and potential utility. 

## 2. Survey of New Methodology

### 2.1. 1,2,3,4-Tetrahydroquinolines

Tetrahydroquinolines have long been important synthetic targets for chemists due to their ubiquitous distribution in natural products and medicinal agents [1,5,19,20,21,22]. Because it is an important structural motif in a large number of biologically active compounds, many synthetic schemes have been developed to prepare them. Domino reactions have proven to be particularly valuable for generating tetrahydroquinolines bearing previously inaccessible substitution patterns, and thus, many new drugs have been designed around these systems over the past two decades [5,6].

**Figure 1:**
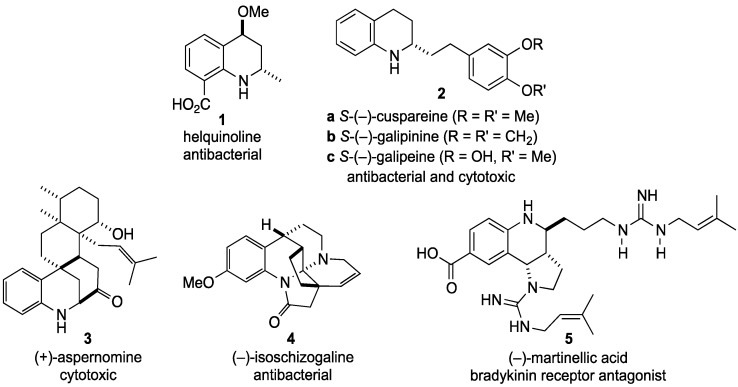
Natural products incorporating tetrahydroquinolines.

Tetrahydroquinolines embedded in natural product structures range from simple to complex. For example, helquinoline (**1**) is a relatively simple molecule with significant antibiotic properties [23]. Similarly, cuspareine (**2a**) and related compounds **2b** and **2c** have shown antibacterial as well as cytotoxic activity [24]. More complex systems include (+)-aspernomine (**3**), a potent cytotoxic agent [25] and (−)-isoschizogaline (**4**), a potentially useful antibiotic [26]. Finally, (−)-martinellic acid (**5**) is known to be a non-peptide antagonist for the bradykinin B_1_ and B_2_ receptors [27] (Figure 1).

**Figure 2:**
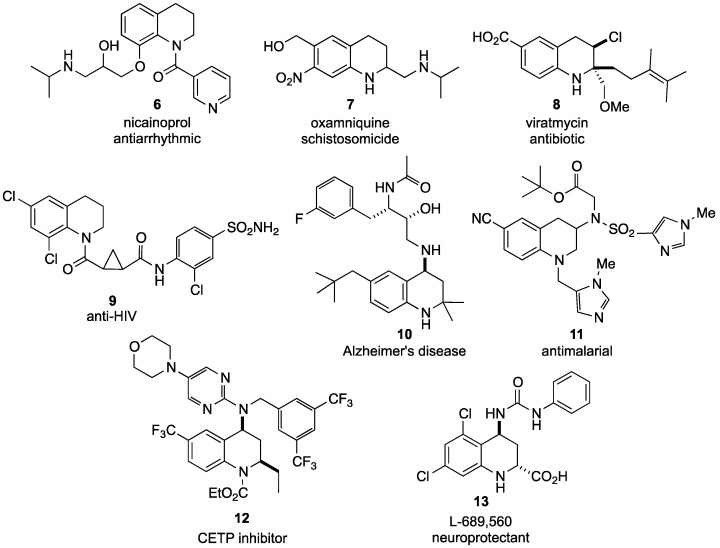
Drugs incorporating tetrahydroquinolines.

The 1,2,3,4-tetrahydroquinoline nucleus is a prevalent core structure in a myriad of synthetic pharmaceuticals as well. Nicainoprol (**6**) is an antiarrhythmic drug [28], oxamniquine (**7**) is a schistosomicide [29], and virantmycin (**8**) is an antiviral antibiotic that also possesses antifungal activity [30,31,32,33]. Additionally, compound **9** is being evaluated for use in the treatment of HIV [34], compound **10** is garnering attention as an agent to slow the onset of Alzheimer’s disease [35,36,37], and compound **11** is currently undergoing testing as an antimalarial agent [38]. Furthermore, compound **12** has demonstrated activity as a cholesterol ester transfer protein (CETP) inhibitor and may prove useful for ameliorating hypercholesterolipidemia [39,40], while L-689,560 (**13**) is a neuroprotective agent with potential to minimize ischemic nerve damage following a stroke or heart attack [41] (Figure 2).

Beyond the structures depicted above, additional drug candidates incorporating tetrahydroquinolines have included analgesics [42], anticonvulsants [43], antidepressants [44,45,46], antipsychotics [47], antihypertensives [48,49,50,51], antiarrhythmics [52], antiallergenics [53], antimalarials [54], antitumor [55], anticancer [56], antifungal [57], antichagasic [58], and antiosteoporotics [59]. Other derivatives have exhibited significant immunosuppressant properties sparking studies of their potential as anti-rejection drugs [60,61] and their inhibitory activity toward AMP activated protein kinase has attracted clinical interest as possible treatments for diabetes [62]. With such a wealth of pharmaceutical potential, it is no wonder that tetrahydroquinolines have been frequent targets for new synthetic methods. 

**Scheme 1:**
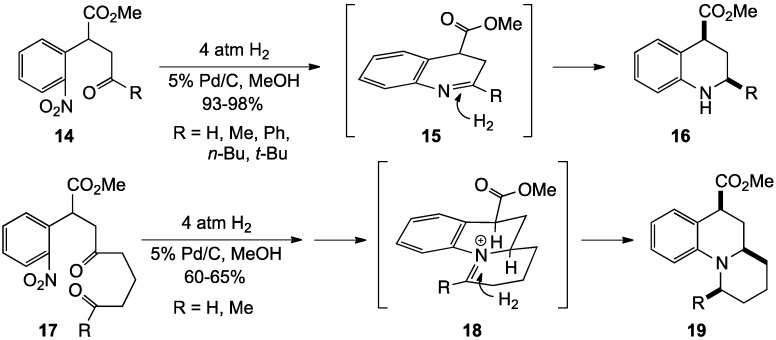
A domino reduction-reductive amination sequence.

Over the past decade, Bunce and co-workers have introduced several practical and efficient domino reactions to generate tetrahydroquinolines. One of these reported the conversion of 2-nitroarylketones and aldehydes to the target heterocycles using a reduction-reductive amination strategy under hydrogenation conditions with 5% Pd/C as catalyst [8]. This synthesis (Scheme 1) featured a multi-step sequence triggered by catalytic reduction of the nitro group in **14**, followed by formation of cyclic imine **15**, and further reduction to yield the tetrahydroquinolines **16** in 93%–98% yield. Reduction of the penultimate imine **15** was highly diastereoselective, giving hydrogen addition to the molecular face opposite the C4 ester group, resulting in a cis relationship between the C2 alkyl and C4 ester in **16**. The authors further observed that addition of formaldehyde to the hydrogenation mixture resulted in the isolation of *N*-methyltetrahydroquinoline derivatives [8]. Building on this strategy, these researchers also developed a reduction-double reductive amination sequence to convert **17** via **18** to the angular-fused tricyclic structures **19** with similar high diastereoselection in 60%–65% yield. One noteworthy finding from the reduction-double reductive amination study was that the process required a relatively large amount of catalyst (>20 wt%), presumably due to poisoning of the catalyst after the initial cyclization. 

**Scheme 2:**
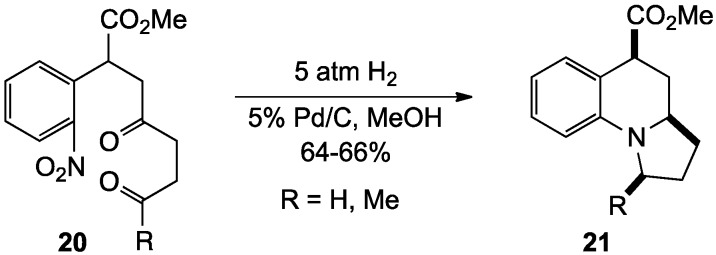
Strained angular fused systems by reduction-double reductive amination.

This same strategy was applied to the ring closure of **20** to give the angular 6-6-5 tricyclic ring structures **21** [63] (Scheme 2). This reaction exhibited the same high diastereoselectivity leading to the all-*cis* product. Unlike the less strained system above, however, this cyclization was found to be pressure dependent, with lower pressures of hydrogen (1–2 atm) giving by-products resulting from incomplete cyclization and higher pressures (5 atm) affording the desired targets in 64%–66% yield. 

**Scheme 3:**
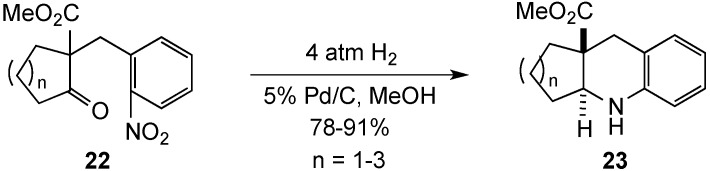
Linear fused systems by reduction-double reductive amination.

Encouraged by this initial success, the Bunce group pursued a similar ring closure to form linear tricyclic systems [64]. Substrates **22** were prepared in one-step and subjected to reductive cyclization using hydrogen and 5% Pd/C to give tetrahydroquinolines **23** in high yield (78%–91%) and with excellent selectivity (>98%) for the trans-fused products (Scheme 3). Thus, as observed in the previous cyclizations, the bridgehead ester sterically directed the addition of hydrogen to the opposite face of the molecule resulting in a *trans* ring junction. Repeating these ring closures with substrates lacking the ester function led to mixtures of *cis*- and *trans*-fused products with a slight preference for the *cis*. 

**Scheme 4:**
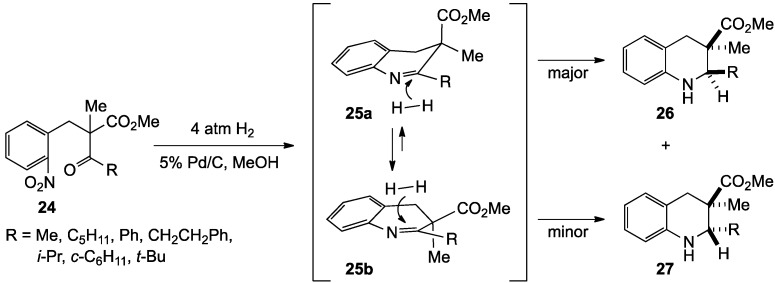
A reduction-reductive amination sequence.

Additional understanding of the stereoselectivity in these ring closures was sought by moving the ester from the α to the β carbon relative to the aromatic ring and introducing a sterically demanding methyl substitutent geminal to the ester [65]. A two-step synthesis furnished precursors **24**, which on reductive cyclization, produced **26** and **27**, respectively, in 78%–87% yield (Scheme 4). While the product having the C2 alkyl *cis* to the C3 ester still predominated in ratios of 6:1 to 16:1, the greater conformational flexibility about the β carbon in these substrates diminished the absolute *cis* selectivity observed in substrates bearing the ester group α to the aromatic ring. An apparent conformational preference for a pseudoaxial ester in **25a** was invoked to rationalize the bias toward the *cis* product in this reaction. 

**Scheme 5:**
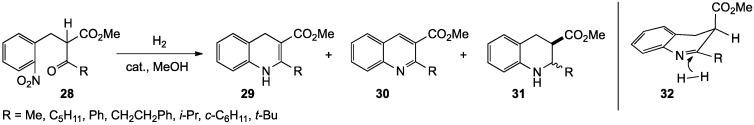
Catalyst and steric shielding effects in the reduction-reductive amination.

A subsequent study examined ring closures in related substrates **28**, bearing an ester at the β carbon with the geminal methyl replaced by hydrogen [66]. Interestingly, this reaction proved to be highly catalyst dependent. Hydrogenation of **28** at 1 atm using 5% Pd/C afforded primarily dihydroquinoline **29**, while at 4 atm, quinoline **30** was the major product. By changing the catalyst to 5% Pt/C (4 atm H_2_), however, the double bond migration and aromatization pathways were dramatically reduced, and formation of tetrahydroquinoline **31** dominated with the *cis* isomer favored by ≥13:1 over the trans. Contrary to expectation, larger R groups gave higher *cis* selectivity for the substituents in the isolated heterocycles. To rationalize this observation, a steric interaction between R and the ester was assumed in the intermediate imine. This would oblige the ester to adopt a pseudoaxial orientation, as in **32**, which would direct hydrogen addition from the distal face of the molecule to generate the *cis* product (Scheme 5). These results demonstrated the importance of catalyst and steric shielding on the reaction outcome. 

**Scheme 6:**
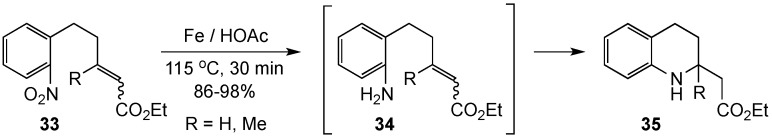
A reduction-Michael addition sequence.

Domino processes initiated by dissolving metal reductions were also investigated as a strategy for synthesizing tetrahydroquinolines [67]. Substrates **33** underwent reduction with iron powder in acetic acid to yield anilines **34**, which were captured in a favorable 6*-exo-trig* Michael addition with the side chain acrylate moiety to afford **35** in 86%–98% yield (Scheme 6). The geometry of the double bond and hindrance at the Michael terminus had a minimal impact on the cyclization process.

**Scheme 7:**
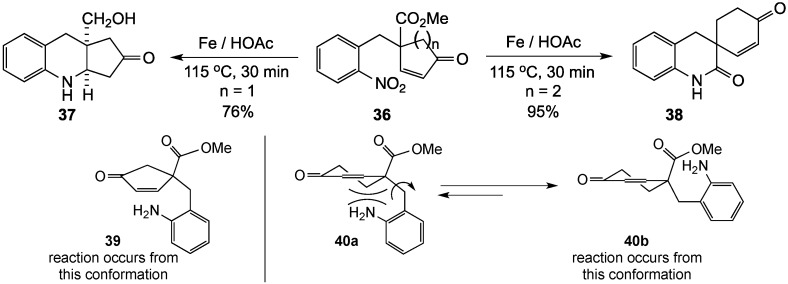
Divergent reactivity based on ring size.

In an extension of this study, divergent reactivity was observed for the reduction-Michael reaction involving 4-(2-nitrobenzyl)-2-cycloalken-1-one systems **36** [68]. In this project, two substrates, differing only in the Michael acceptor ring size, were prepared and reacted with iron in acetic acid. Reduction of the nitro group, followed by conjugate addition of the resulting aniline to the enone moiety, proceeded predictably for the cyclopentenone precursor to give the linear tricyclic system **37** in 76% yield. Somewhat surprisingly, the bridgehead ester of the expected product was also reduced during this reaction. The cyclohexenone substrate, on the other hand, produced the spirocyclic amide **38** as the only product in 95% yield. These results were attributed to the difference in strain as well as alignment of the reacting centers in the two systems. It was reasoned that the more reactive five-membered ring would adopt conformation **39**, which positions the aniline nitrogen to add to the enone double bond. The six-membered ring, on the other hand, would assume conformation **40b** to avoid steric interaction of the amino function with the C5 ring carbon (*viz*. **40a**) resulting in preferential closure on the ester group to give the amide (Scheme 7).

**Scheme 8:**
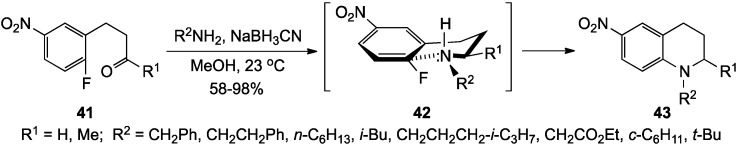
A reductive amination-S_N_Ar reaction.

In a subsequent investigation, Bunce and co-workers designed a system to gauge the feasibility of a domino reductive amination-nucleophilic aromatic substitution (S_N_Ar) sequence for the preparation of tetrahydroquinolines [69,70]. From a mechanistic standpoint, it was anticipated that an initial reductive amination to the side chain carbonyl group would yield an amine, which would undergo S_N_Ar ring closure with the activated aromatic acceptor. In practice, the reaction proved highly successful and produced the desired heterocycles in 58%–98% yields. Ketones (e.g., **41**, R^1^ = Me) furnished superior yields in comparison to aldehydes (R^1^ = H) due to their increased stability toward the reaction conditions and because of steric interactions in a proposed chair-like conformation for ring closure. According to this hypothesis, the α methyl group in amine **42** (R^1^ = Me; R^2^ = alkyl), produced from **41**, should adopt a pseudoequatorial orientiation, which would juxtapose the reactive sites and promote the ring closure to **43** (Scheme 8). On the other hand, an aldehyde-derived amine would be expected to exhibit a more random conformation that would make the cyclization less favorable. The limitation of this process is associated with the S_N_Ar ring closure, as it will proceed only if there is an electron-withdrawing group, such as NO_2_, at C4 (or C2) relative to the fluorine.

**Scheme 9:**
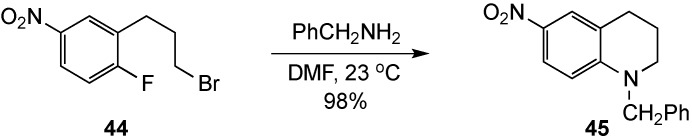
An S_N_2-S_N_Ar reaction

Bunce and co-workers continued to exploit the S_N_Ar terminating event in a domino S_N_2-S_N_Ar tetrahydroquinoline synthesis [10,71]. Specifically, this process involved an intermolecular S_N_2 reaction of benzylamine with the primary side chain bromide in **44**, followed by an intramolecular S_N_Ar displacement of fluoride from the activated aromatic ring. The reaction proceeded in DMF at ambient temperature to afford a 98% yield of **45** (Scheme 9). Unfortunately, only one case of this transformation was reported, and thus, the full potential of the process has not been established.

**Scheme 10:**
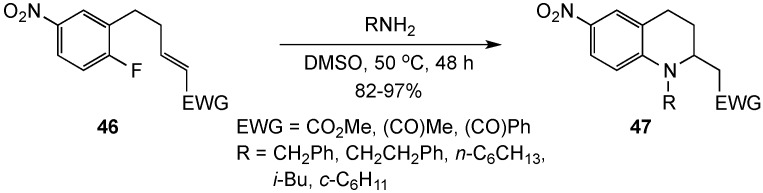
An S_N_Ar-Michael sequence.

Assembly of tetrahydroquinolines by consecutive additions to a 4-nitrofluorobenzene and a Michael acceptor in **46** to give **47**, was also investigated by the Bunce group [71] (Scheme 10). The reaction gave 82%–97% yields for unhindered amines but was sensitive to steric bulk α to the amine nitrogen. Heterocyclization could conceivably occur by an S_N_Ar addition to the 1-fluoro-4-nitrobenzene, followed by Michael reaction with the side chain acrylate or by the reverse sequence. The reaction chronology was inferred by competitive reaction of benzylamine with separate mixtures of 2-fluoro-5-nitrotoluene with methyl (*E*)-5-phenyl-2-pentenoate and (*E*)-1,5-diphenyl-2-penten-1-one at 50 °C. For the unsaturated ester, preferential reaction occurred with the nitroarene suggesting an S_N_Ar-Michael sequence, while the ketone (a better Michael acceptor) was less conclusive, showing only a slight preference for the S_N_Ar reaction.

**Scheme 11:**
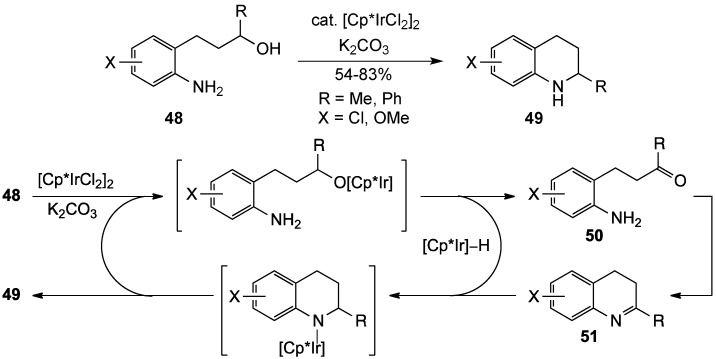
A metal-catalyzed oxidative cyclization of amino alcohols.

In 2002, the Fujita group unveiled a new metal-catalyzed oxidative cyclization of amino alcohols **48** to tetrahydroquinolines **49** in the presence of dichloro(pentamethylcyclopentadienyl)iridium(III) dimer and base [9]. Although the mechanism was unclear, it was postulated that the iridium complex acted on **47** to catalytically oxidize the alcohol to aldehyde **50**. This aldehyde was then captured by the proximal amino group to form imine **51**, which was reduced to the tetrahydroquinoline **49** by the hydrido iridium species produced during the oxidation (Scheme 11). This reaction was also extended to structures bearing a two-carbon side chain, where an alternative mechanistic scenario was proposed to give indoles. 

**Scheme 12:**
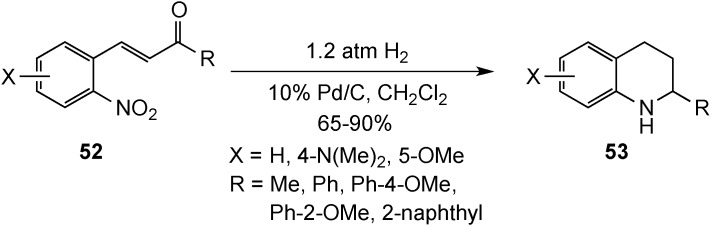
Reductive cyclization of 2-nitrochalcones.

In 2010, Patti and Pedotti published a simple method for converting 2-nitrochalcones **52** to tetrahydroquinolines **53** *via* a reductive cyclization under catalytic hydrogenation conditions [72] (Scheme 12). Rapid reduction of the side chain double bond, in addition to the nitro group, was essential to prevent the formation of quinoline by-products. The solvent reportedly played a crucial role in this hydrogenation process, with dichloromethane affording the best selectivity and highest yields (65%–90%). Operational simplicity, excellent atom economy, and low H_2_ pressures were advantages of this process, but the required use 10 wt% of 10% Pd/C was a drawback.

**Scheme 13:**
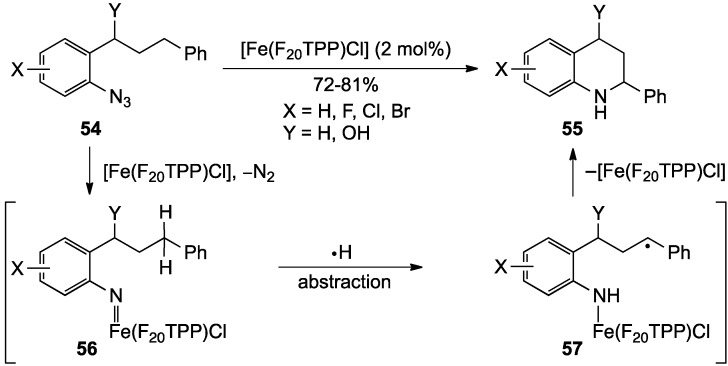
A metal-mediated heterocyclization of aryl azides.

Che and co-workers recently disclosed a metal-mediated heterocyclization of **54** to **55** involving an intramolecular nitrene C-H insertion process promoted by the commercially available, air-stable [Fe(III)(F_20_TPP)Cl] complex [F_20_TPP = *meso*-tetrakis(pentafluorophenyl)porphyrinato dianion] [73]. The process offered a one-step route to a limited selection of 2-aryl-1,2,3,4-tetrahydroquinolines in 72%–81% yields. The proposed mechanism involved sequential formation of iron-nitrene complex **56**, abstraction a side chain γ-hydrogen to give benzylic radical **57**, and cyclization to product **55** (Scheme 13). 

**Scheme 14:**
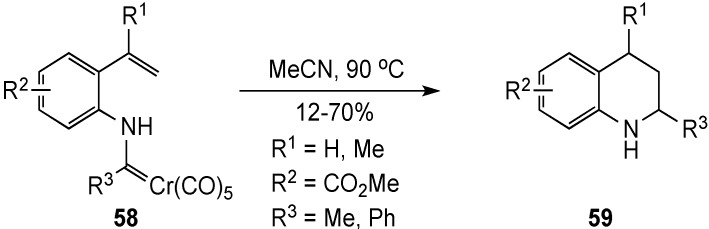
Thermal cyclizations of chromium Fischer carbenes.

Söderberg *et al.* have applied a novel thermal annulation reaction of *N*-(2-alkenylphenyl)amino-substituted chromium Fischer carbenes **58** to synthesize tetrahydroquinolines **59** [74] (Scheme 14). The extent of product formation was found to be dependent on the solvent as well as steric and electronic factors in the substrates. Yields were highest when acetonitrile was the solvent, but the results were highly variable (12%–70%) and no preferred substitution pattern favoring heterocycle formation emerged from the study. The major by-products were quinoline and 2-aminostyrene.

**Scheme 15:**
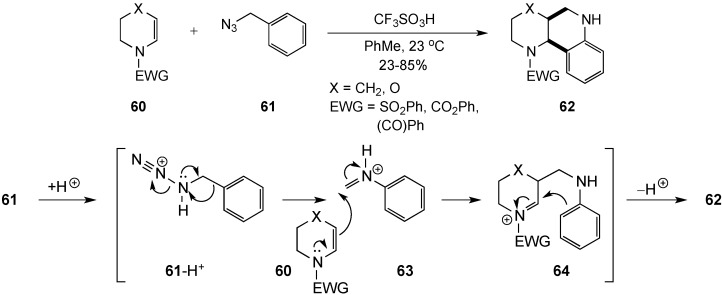
Cyclization of enamides with benzyl azide.

Gigant and Gillaizeau reacted enamides **60** with benzyl azide **61** under acid conditions in a novel synthesis of fused-ring tetrahydroquinolines **62** [12]. In this scheme, treatment of the reactants with triflic acid spurred rearrangement of **61** to give an *N*-phenyliminium intermediate **63**. Subsequent nucleophilic addition of **60** to this iminium species gave **64**, which cyclized to the desired heterocycles **62** (Scheme 15). The sequence provided the products in 23%–85% yields with complete cis diastereoselectivity. The scope of the reaction was further expanded to include the use of 5,6-benzo-fused enamides, which produced tetracyclic products. 

**Scheme 16:**
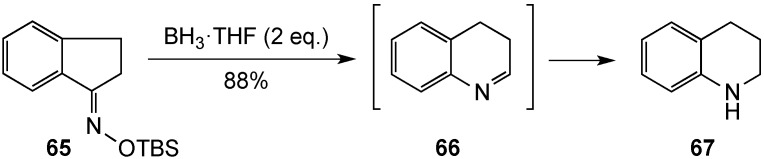
Tetrahydroquinoline from 1-indanone *O*-TBS oxime.

Ortiz-Marciales and co-workers have documented a simple protocol to convert 1-indanone *O*-TBS oxime (**65**) to tetrahydroquinoline (**67**) using 2 eq. of borane-tetrahydrofuran complex [75]. A mechanism was not offered, but likely involved carbon-nitrogen double bond reduction in **65**, followed by formal α-elimination of TBSOBH_2_ with aryl migration to the electron-deficient nitrogen to generate the six-membered cyclic imine **66** (Scheme 16). Various borohydrides in the mixture would then reduce the imine double bond and yield the product. Unfortunately, this study reported only one example of this process leading to the unsubstituted derivative **67**, and thus, the generality of the process is unclear.

**Scheme 17:**
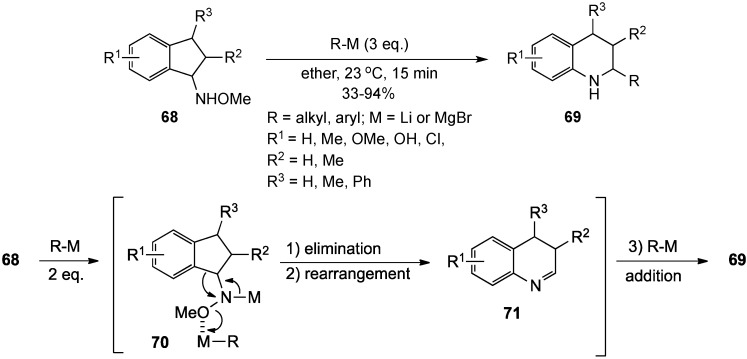
Tetrahydroquinolines from *N*-indanyl(methoxy)amines.

The Miyata group advanced a related option for the conversion of *N*-indanyl(methoxy)amines **68** to 2-substituted tetrahydroquinolines **69** by treatment of the former with 3 eq. of an organomagnesium or organolithium reagent [16]. The route involved a domino sequence initiated by deprotonation of the nitrogen and coordination of an organometallic species with oxygen to give **70**. Rearrangement of the resulting arylmethylamide with loss of methoxide to generate the ring-expanded imide **71**, and addition of a molecule of the organometallic reagent to the imide would then afford product **69** (Scheme 17). The reaction generally required electron-donating groups on the aromatic ring and achieved yields in the 33%–94% range. The lowest yields were observed when R^3^ was methyl or phenyl. The proposed mechanism for this rearrangement is formulated below. 

**Scheme 18:**
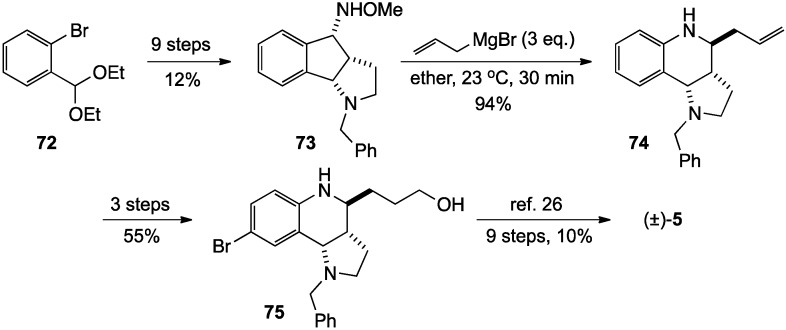
Synthesis of (±)-martinellic acid.

These same authors validated the utility of this reaction by exploiting it in a stereoselective formal synthesis of the alkaloid (±)-martinellic acid (**5**). The key intermediate **73** was prepared from bromoacetal **72** in 9 steps (12% yield, 34% based on recovered starting material). Treatment of **73** with 3 eq. of allylmagnesium bromide in ether then gave **74** in 94% yield. This intermediate was subsequently elaborated in three steps (55% yield) to relay structure **75**, which had been previously converted to (±)-**5** [27] (Scheme 18).

**Scheme 19:**
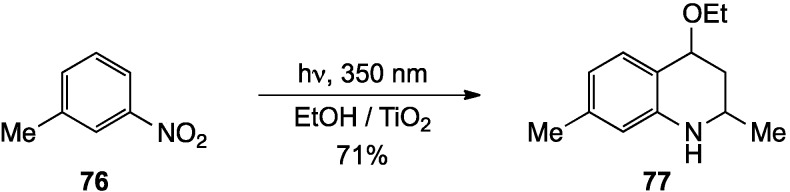
A photochemical synthesis of tetrahydroquinolines.

Finally, a photochemical approach to tetrahydroquinolines involving irradiation of an ethanol solution of 3-methyl-1-nitrobenzene (**76**) at 350 nm in the presence of TiO_2_ was reported by Park and co-workers [76]. Under these conditions, it was proposed that TiO_2_ catalyzed reduction of **76** to the aniline and simultaneous oxidation of ethanol to acetaldehyde. In ethanol solvent, some of the acetaldehyde likely existed as the diethyl acetal, which upon loss of ethanol, would give ethyl vinyl ether. Addition of this vinyl ether to the imine derived from 3-methylaniline and acetaldehyde, would then deliver 4-ethoxy-2,7-dimethyl-1,2,3,4-tetrahydroquinoline (**77**) (Scheme 19). Although the yield was respectable (71%), the reaction scope was not addressed in this report, and thus, the generality of the process remains unknown.

### 2.2. 2,3-Dihydro-4(1H)-quinolinones

**Figure 3:**
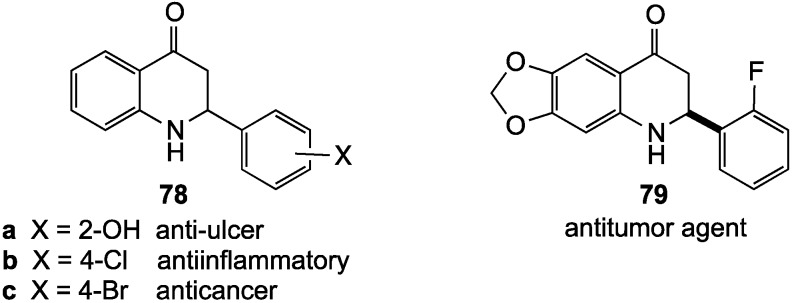
Drugs incorporating 2,3-dihydro-4(1*H*)-quinolinones.

2,3-Dihydro-4(1*H*)-quinolinones can essentially be thought of as aza-analogs of flavanones. While many of these derivatives are intermediates in syntheses of 4(1*H*)-quinolinones [77], some express medicinal properties of their own [78]. Most drugs possessing this pharmacophore are simple 2-aryl-substituted derivatives such as **78** [79,80] and **79** [81] (Figure 3). Other dihydroquinolinones, with more diverse substitution, are under consideration as treatments for high blood pressure [82,83], pain [84] and Alzheimer’s disease [85,86]. One patent also asserts that several members of this compound family are useful as pesticides [87].

**Scheme 20:**
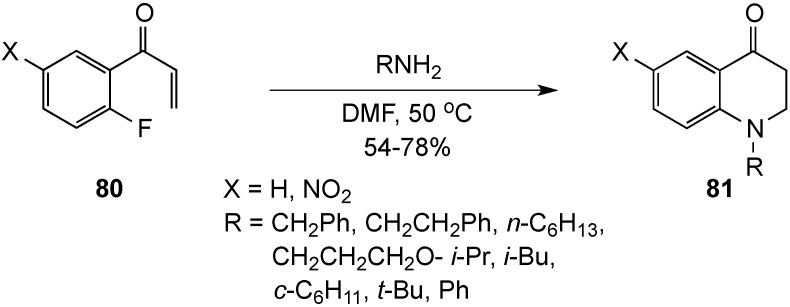
A domino Michael-S_N_Ar approach.

A concise and straightforward preparation for *N*-alkyl-2,3-dihydro-4(1*H*)-quinolinones has been pioneered by Bunce and co-workers using a domino Michael-S_N_Ar approach [88]. The requisite 1-aryl-2-propen-1-one derivatives **80**, which incorporated both Michael and S_N_Ar acceptors, were efficiently accessed in two steps. Treatment of these substrates with a selection of primary amines afforded the dihydroquinolinones **81** in 54%–78% yield by Michael addition to the enone, followed by S_N_Ar ring closure (Scheme 20). One drawback to the method was that the final ring closure did not tolerate the presence of electron-donating groups on the S_N_Ar acceptor ring. Additionally, though nitro activation was not essential to the process, yields were diminished in less activated systems when the incoming amine was branched α to the nitrogen.

**Scheme 21:**
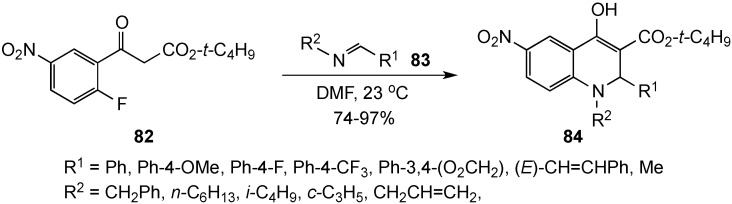
An imine addition-S_N_Ar sequence.

Bunce and Schammerhorn have described a new domino strategy for the synthesis of highly substituted 2,3-dihydro-4(*1H*)-quinolinones using an imine addition-S_N_Ar approach [89]. Reaction of *tert*-butyl 2-fluoro-5-nitrobenzoylacetate (**82**) with pre-formed imines **83** at room temperature furnished the target compounds **84** in a single operation. The annulations were successful with aldimines, but failed to yield the desired targets using more sterically demanding ketimines. The sequence proceeded by addition of ketoester **82** to imine **83**, followed by S_N_Ar reaction of the resulting secondary amine to furnish the dihydroquinolinones as the stable enols **84** in 74%–97% yields (Scheme 21). Base was not required for the reaction to occur. Precursors with methyl and ethyl esters gave comparable results, but *tert*-butyl esters were deemed more versatile for subsequent transformations. 

**Scheme 22:**
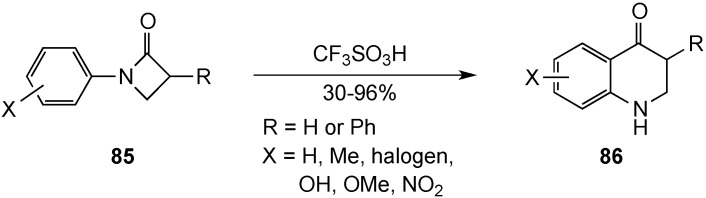
A Fries-like rearrangement of *N-*arylazetidin-2-ones.

A Fries-like rearrangement of *N-*arylazetidin-2-ones **85** promoted by triflic acid formed the basis of a synthesis of 2,3-dihydro-4(*1H*)-quinolinones **86** reported by Banwell and co-workers [11] (Scheme 22). This procedure is a modification of an earlier report by Kano *et al.* [90], which utilized refluxing trifluoroacetic acid as the solvent/catalyst. The use of triflic acid, however, permitted reactions at room temperature with greater product returns. Yields were generally in the 30%–96% range, but lower when electron-withdrawing substituents were positioned on the aromatic ring. Two earlier papers by Anderson and Tepe reported this same reaction in 1,2-dichloroethane with fewer examples but similar results [91,92]. A disadvantage of these recent studies was the high cost of the azetidin-2-one used to synthesize the substrates. The initial report by Kano [90] and one by Gomtsyan [93] gave more economical preparations.

**Scheme 23:**
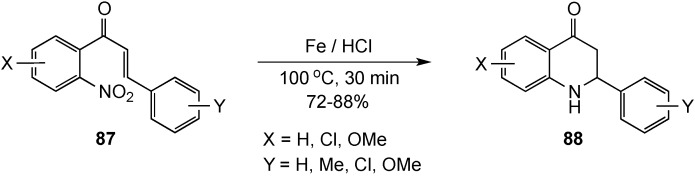
A dissolving metal reduction-cyclization reaction.

A protocol to access 2-aryl-2,3-dihydro-4(1*H*)-quinolinones in 72%–88% yield *via* a dissolving metal reduction-cyclization sequence has also appeared [94]. The domino sequence was initiated by reduction of the nitro group of **87** using iron powder in acids of varying strength. A series of experiments revealed that iron in concentrated HCl at 100 °C for 30 min afforded superior results, suggesting that strong acid is the key to efficient conversions. Following reduction of the nitro group, cyclization to **88** presumably involved addition of the aniline amino group to the protonated enone (Scheme 23).

**Scheme 24:**
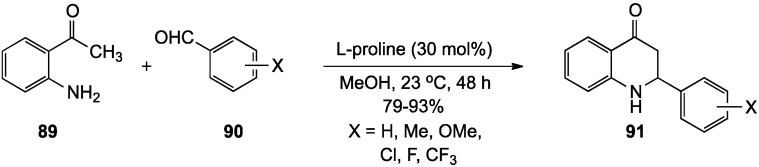
A proline-catalyzed cyclization of 2-aminoactophenone and benzaldehydes.

The Chandrasekar group explored a possible asymmetric route to 2-aryldihydroquinolinones from 2-aminoacetophenone (**89**) and benzaldehydes **90** using l-proline as a catalyst at room temperature [95]. Although the desired products **91** were isolated in 79%–93% yields, the observed ees were <10%. An advantage of this method was that the reaction proceeded with both electron-donating and electron-withdrawing X groups on the aldehyde reacting partner (Scheme 24).

**Scheme 25:**
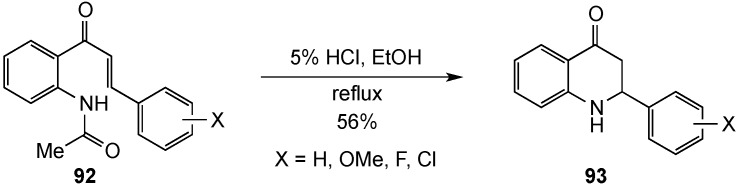
Domino hydrolysis-cyclization of acetamidochalcones.

The Nepali group offered an alternative synthesis of similar structures from acetamidochalcones **92** [77]. The reactions proceeded by sequential hydrolysis of the amide, followed by Michael addition to the unsaturated ketone to give the 2,3-dihydroquinolinones **93** (Scheme 25). Although 15 cases were reported, only one yield of 56% was recorded for **93** (X = H) since the intended aim of this study was the synthesis of the corresponding 4(1*H*)-quinolinones. The ring closure likely proceeded as described for the previous reaction. 

**Scheme 26:**
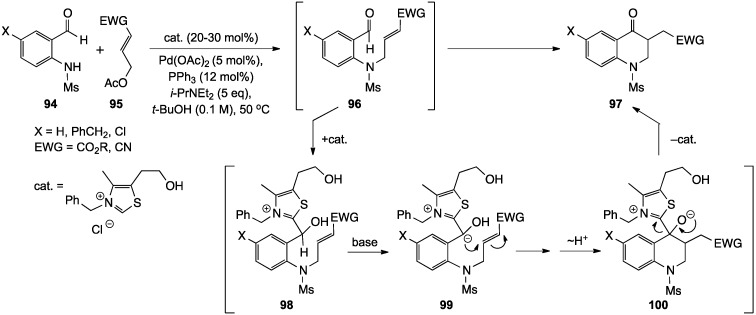
A multi-catalytic approach to 2,3-dihydro-4(1*H*)-quinolinones.

Hamada and co-workers have described an interesting multi-catalytic approach to 3-substituted 2,3-dihydro-4(1*H*)-quinolinones [96]. This process involves sequential Pd-catalyzed *N*-alkylation of 2-methanesufonamidobenzaldehydes **94** with allylic acetates **95** to give aldehyde **96**, followed by intramolecular thiazolium salt-catalyzed Stetter reaction between the aldehyde in **96** and the side chain Michael acceptor to generate heterocycle **97**. The Stetter reaction is a variant of the well-known thiazolium-catalyzed benzoin condensation, where the intermediate anion **99** is captured by 1,4-addition to an unsaturated system rather than 1,2-addition to a carbonyl. The mechanism, applied to the conversion of **96** to **97**, is outlined below (Scheme 26). The one-flask domino version of the reaction was examined for a small selection of compounds, but the results were erratic, with several reactions giving near quantitative yields and others failing completely. Thus, further work is necessary to fully elucidate and optimize this process.

**Scheme 27:**
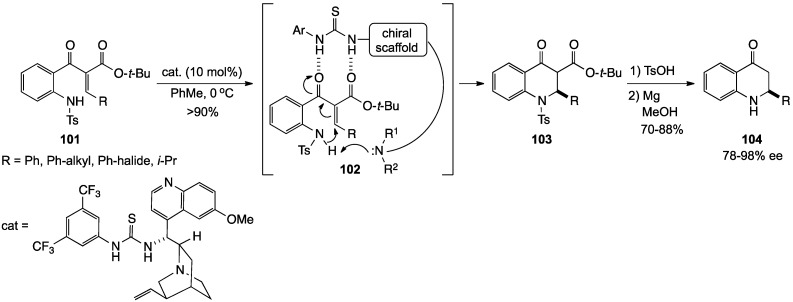
The Lu asymmetric synthesis of 2,3-dihydro-4(1*H*)-quinolinones.

Lu and co-workers presented an elegant approach to asymmetric 2-aryl-2,3-dihydro-4(1*H*)-quinolinones using a chiral tertiary amine base tethered to a bifunctional thiourea derivative [97]. In this reaction, a thiourea catalyst, derived from quinine, initially hydrogen-bonded with the β-ketoester moiety of substrate **101** to give **102**. The basic quinuclidine moiety of the quinine then deprotonated the acidic sulfonamide function, and the heterocyclic ring closed by conjugate addition to the highly polarized side chain double bond to give **103**. Ester hydrolysis and decarboxylation, followed by cleavage of the sulfonamide then delivered the chiral dihydroquinolinones **104**. Overall yields for the sequence were 65%–91% with 78%–98% ee (Scheme 27).

**Scheme 28:**
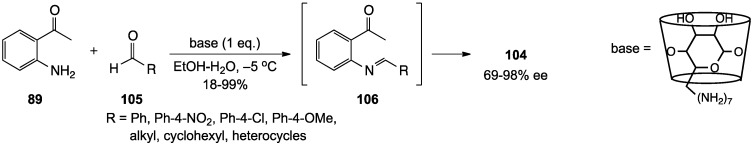
The Pitchumani asymmetric synthesis of 2,3-dihydro-4(1*H*)-quinolinones.

A complementary protocol for the synthesis of chiral dihydroquinolinones has been described by Pitchumani and Kanagarj [98]. The target heterocycles were produced from 2-aminoacetophenone (**89**) and aldehydes **105** in aqueous ethanol using stoichiometric per-6-amino-β-cyclodextrin (per-6-ABCD) as the base and chiral supramolecular host. The conversion occurred by initial condensation of **89** with **105** to give imines **106**, followed by deprotonation of the methyl ketone by the per-6-ABCD and closure on the carbon-nitrogen double bond to give 2,3-dihydro-4(1*H*)-quinolinones **104** (Scheme 28). The reaction was performed using a diverse selection of aldehydes bearing electron-rich and electron-poor aromatic as well as alkyl, cycloalkyl and heterocyclic R groups. The reported yields ranged from 18%–99%, and the products were obtained with 69%–98% ee. A major strength of this procedure was that the base was easy to prepare and could be recovered and reused without loss of activity. 

### 2.3. 4(1H)-Quinolinones

**Figure 4:**
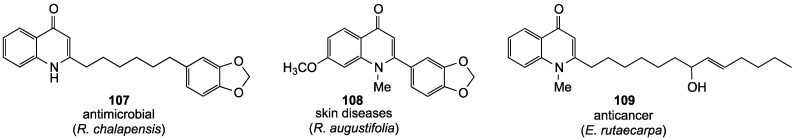
Natural products incorporating 4(*1H*)-quinolinones.

The 4(*1H*)-quinolinones, also known as 4-quinolones, are a class of naturally occurring compounds, which display pharmacological activity for mitigating the symptoms of innumerable diseases and conditions [99,100]. Quinolinone alkaloids are present in many plants that were used as local remedies in ancient times. For example, compound **107**, originally employed in the Middle East as an antirheumatic and a treatment for snakebites, displays significant antimicrobial activity [101]. Compound **108**, a traditional skin medication from Indonesia, expresses CYP2D6 inhibitory activity that can affect the metabolism of drugs in the body [102]. Finally, compound **109**, used as a Chinese folk remedy for headache and acid reflux, has recently shown broad anti-inflammatory, anticancer, and antimycobacterial properties [103,104,105,106] (Figure 4).

**Figure 5:**
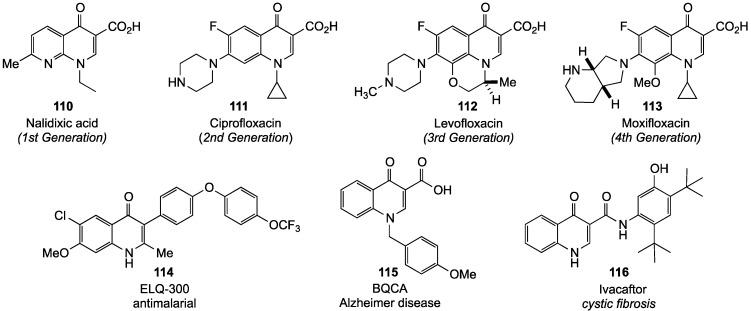
Drug compounds incorporating 4(*1H*)-quinolinones.

Numerous 4(1*H*)-quinolinones are also found as prominent substructures in hundreds of drugs including antibiotics [107], antimalarials [54,108], antihypertensives [109], anticancer agents [110,111], antivirals [112] and compounds to slow the progression of Alzheimer’s disease [113,114]. Nalidixic acid (**110**), a 1st generation quinolinone antibiotic has value for the treatment of urinary bacterial infections [100]. Due to its broad spectrum activity against both Gram-negative and Gram-positive bacteria, related 2nd, 3rd and 4th generation antibiotics (more than 32 compounds), modified to contain a central fluoroquinolinone core, have been developed commercially [115]. The most prominent member of the 2nd generation drugs is ciprofloxacin (**111**), which exhibits powerful activity against many bacterial pathogens [116,117]. Further structural modification in the floxacin series gave chiral 3rd and 4th generation antibiotics **112** and **113**, respectively, which are potent against a broad spectrum of bacterial strains with fewer side effects [118,119]. Fluoroquinolinones not only express potent antibiotic activity in humans, but also serve as antimicrobial and a bactericidal agents for veterinary applications [120]. Several other drugs based on the 4(1*H*)-quinolinone system include ELQ-300 (**114**), an antimalarial drug [108], BQCA (**115**), a promising medication to alleviate the effects of Alzheimer’s disease [113], and Ivacaftor (**116**), a drug used for the treatment of cystic fibrosis [121,122] (Figure 5).

**Scheme 29:**
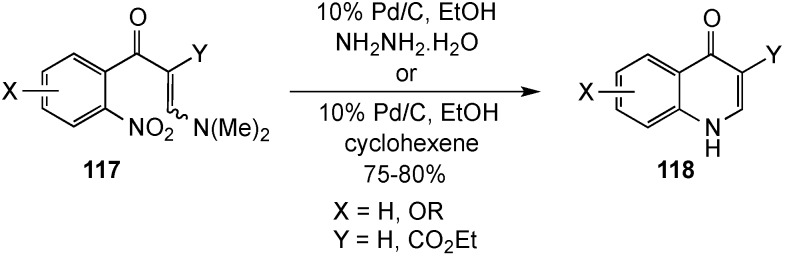
A reduction-addition-elimination sequence.

Two groups have developed syntheses of 4(1*H*)-quinolinones from enaminones such as **117**, which were available *via* a short synthesis [123,124]. Reduction of the nitro function in **117**, using 10% Pd/C in ethanol containing hydrogen donors such as hydrazine hydrate or cyclohexene, initiated a domino process involving Michael addition of the amino group to the polarized double bond, followed by elimination of dimethylamine to provide heterocycles **118** in 75%–80% yields (Scheme 29). This addition-elimination strategy, known as the Leimgruber-Batcho reaction, has been previously used for the synthesis of indoles, but has proven equally valuable for the preparation of 4(1*H*)-quinolinones. The reaction proceeded cleanly for both singly and doubly activated side chain Michael systems.

**Scheme 30:**
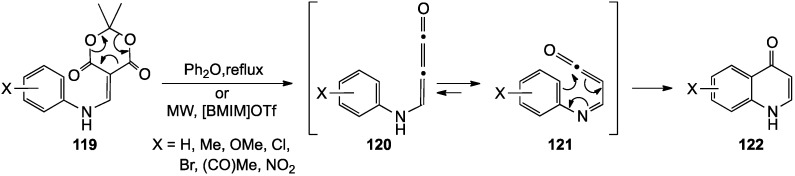
4(1*H*)-Quinolinones from 2-anilinomethylene derivatives of Meldrum’s acid.

In 1983, McNab and co-workers [125] reported a synthesis of 4(1*H*)-quinolinone in 90% yield from the 2-anilinomethylene derivative of Meldrum’s acid **119** under flash vacuum pyrolysis conditions at 600 °C and 10^−2^ torr. The suggested mechanism proceeded through a 3-anilino-1,2-propadien-1-one intermediate **120**, which tautomerized to the iminoketene **121**, cyclized, and rearomatized to give the quinolinone **122** (Scheme 30). Valderrama [13] and Huang [15] independently expanded this domino approach to prepare a number of substituted 4(*1H*)-quinolinones with electron-donating and electron-withdrawing substituents on the aromatic ring. These investigators accomplished the conversion to quinolinones in yields of 54%–96% under thermolysis conditions in boiling diphenyl ether (15–20 min, 250–260 °C). A later paper by the Al-Awadi group [126] further studied this transformation for a series of substrates under flash vacuum and static pyrolysis conditions. Al-Awadi noted that cyclizations under these conditions were strongly influenced by aryl substitution, with nitroarenes giving poor yields in contradiction to one result in the Huang paper [15]. Finally, this same cyclization was performed by Yadav and co-workers under microwave irradiation (80 °C, 120 min) in the ionic liquid [BMIM]OTf (1-butyl-3-methylimidazolium trifluoromethanesulfonate) [127]. Employing this protocol, a modest selection of 4(*1H*)-quinolinones was generated in 68%–94% yields. 

**Scheme 31:**
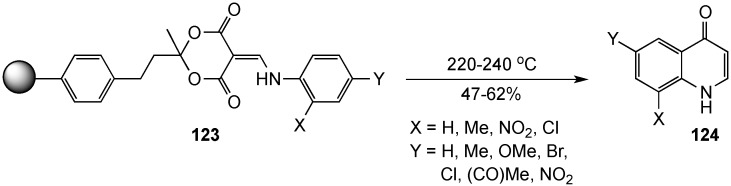
Solid phase organic synthesis of 4(1*H*)-quinolinones.

The Huang group extended their contribution to this area by using a solid phase organic synthesis strategy to prepare 4(1*H*)-quinolinones [14,128,129]. In this approach, the Merrifield resin was synthetically elaborated to attach a Meldrum’s acid moiety to the polymer backbone. Further reaction with triethyl orthoformate, followed by treatment with substituted anilines, then gave the polymer-bound 2-anilinomethylene Meldrum’s acid derivatives **123**. Subsequent thermolysis of these surface-modified polymer substrates in boiling diphenyl ether produced the desired heterocycles **124** in 47%–62% yields (Scheme 31). A practical application of this method is the preparation of drug candidates using a combinatorial strategy. Despite the assertion that the polymer support can be reused, there are limitations to this approach, due to the expense of the resin and the need to reassemble the Meldrum’s acid moiety (three steps, 55%–60%) for each batch.

**Scheme 32:**
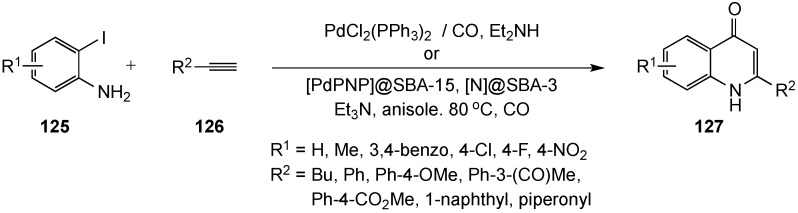
A Sonogashira carbonylative cross-coupling-cyclization reaction.

Kalinin and co-workers have reported a novel approach for the synthesis of 4(*1H*)-quinolinones using a heterocyclic Sonogashira carbonylative cross-coupling-cyclization procedure [17,130]. The reaction was envisioned to involve a 6-*endo-dig* cyclization from an alkynyl ketone formed by a PdCl_2_(PPh_3_)_2_-catalyzed coupling of 2-iodoanilines **125** and terminal alkynes **126** with carbon monoxide to afford quinolinones **127** in 49%–95% yields (Scheme 32). Following the initial disclosure of this reaction, Djakovitch *et al.* reported an intriguing modification of the method. These researchers prepared and tested a reusable heterogeneous catalyst mixture of [PdPNP]@SBA-15 and [N]@SBA-3, which immobilized PdCl_2_(PPh_3_)_2_ and the amine base, respectively, on an Santa Barbara Amorphous (SBA) silica support [131,132]. This catalyst was able to deliver the parent 2-phenyl-4(1*H*)-quinolinone (R^1^ = R^2^ = H) with low Pd contamination in 60%–70% for three cycles, but gradually lost activity thereafter. This strategy merits further development, but currently, the required synthesis of the SBA silica and the multi-step protocols for preparation of the catalysts are barriers to its use. 

**Scheme 33:**
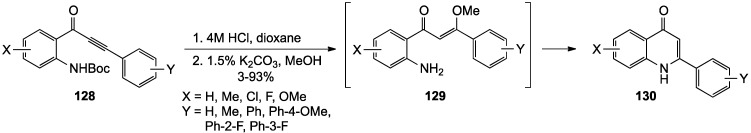
Synthesis of 2-aryl-4(*1H*)-quinolinones from ynones.

The Georg group has documented an alternative scheme [18] for the synthesis of a library of 2-aryl-4(*1H*)-quinolinones using a parallel synthesis approach. In this project, ynone substrates **128** were subjected to HCl in dioxane, followed by K_2_CO_3_ in methanol for a period of 4 days at 50 °C to yield the corresponding products **130** in 3%–93% yields. Although the reaction was time consuming, the process did not require strong base or high temperatures. Mechanistically, treatment of **128** with acid initially resulted in hydrolysis of the Boc group. Methanol then served as a catalytic nucleophile in a domino process triggered by conjugate addition to the triple bond under basic conditions to give **129**. Subsequent to this, the ortho amino group underwent 6*-endo-trig* addition to the double bond, and methanol was eliminated to afford quinolinones **130** (Scheme 33).

**Scheme 34:**
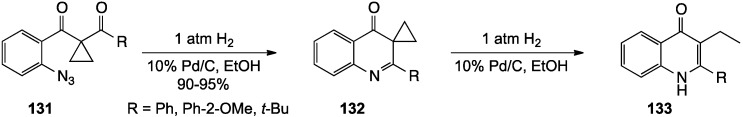
A reduction-cyclization-ring opening strategy.

An interesting reduction-cyclization-ring opening sequence to prepare 4(1*H*)-quinolinones from azido-cyclopropyl ketones has been described by Ren and co-workers [133]. Treatment of aromatic azides **131** with 1 atm of H_2_ over catalytic 10% Pd/C in ethanol resulted in reduction to the aniline and ring closure on the side chain γ carbonyl to give spirocyclopropyl imines **132**. Continued exposure to hydrogenation conditions led to three-ring opening by 1,4-addition of hydrogen to the cyclopropyl imine subunit to give the 2,3-disubstituted quinolinone derivatives **133** (Scheme 34). The reaction was reported for three examples, all of which proceeded in >90% yield. Additional work should expand the range of viable substrates for this sequence and reveal potential applications in synthesis. 

**Scheme 35:**
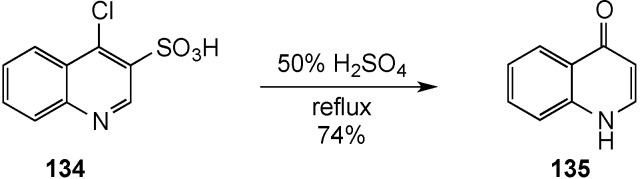
A hydrolysis-desulfonation reaction.

Finally, Skrzypek has communicated a straightforward synthesis of the parent 4(*1H*)-quinolinone by acid hydrolysis of 4-chloro-3-quinolinesulfonic acid (**134**) using 50% H_2_SO_4_ at reflux for 3 h [134]. These conditions eventuated a domino process that includes (1) hydrolysis of the activated chlorine substituent and (2) desulfonation to generate **135** in 74% yield (Scheme 35). Unfortunately, the scarcity of related structures substituted with functionality that can withstand these harsh conditions severely limits the utility of this route. 

## 3. Conclusions

This brief compilation describes a wide range of domino reactions applied to the synthesis of tetrahydroquinolines and related structures over the past 20 years. Efforts are continuing in this active field to create additional approaches to these and many other heterocyclic targets that serve as pharmaceutical precursors. It is likely that future work will dramatically increase the number of accessible derivatives as well as the efficiency and selectivity of these transformations. Application of these processes to the synthesis of previously unknown molecular structures should insure that new, more potent medicinal agents are available to treat drug-resistant pathogens as well as other diseases and conditions that threaten human and animal health. 
